# Psychological distress in patients with serious mental illness during the COVID-19 outbreak and one-month mass quarantine in Italy

**DOI:** 10.1017/S0033291720001841

**Published:** 2020-05-19

**Authors:** Felice Iasevoli, Michele Fornaro, Giordano D'Urso, Diana Galletta, Claudia Casella, Mariano Paternoster, Claudio Buccelli, Andrea de Bartolomeis, the COVID-19 in Psychiatry Study Group

**Affiliations:** 1 Section of Psychiatry, Department of Neuroscience, School of Medicine, University of Naples Federico II, Naples, Italy; 2 Legal Medicine Unit Department of Advanced Biomedical Sciences, School of Medicine, University of Naples Federico II, Naples, Italy; 3 Chair Staff for Health Education and Sustainable Development, UNESCO, Naples, Italy

Several recent reports have investigated the psychological effects of the COVID-19 pandemic on health-care workers (Lai et al., 2020), individuals with COVID-19 (Bo et al., 2020), or the general population (Qiu et al., 2020). However, no study has investigated the effects of the pandemic and mass quarantine on patients with serious mental illness. Notably, Italy has been one of the hardest-hit countries in the world and adopted an extended lockdown of the entire territory. In this context, we conducted an observational, case−control analysis to examine the severity of COVID-19-related perceived stress, anxiety, depressive, and psychotic symptoms in 205 patients with serious mental illness, 51 their first-degree relatives, and 205 non-psychiatric subjects after one-month lockdown.

All participants were from Naples area. All evaluations were carried out from 13 to 17 April, corresponding to one-month quarantine and approximately 50 days from outbreak start in Italy. Patients followed at our unit were contacted by phone by trained clinicians who were already acquainted with them. The following diagnosis (extrapolated from clinical records) were included: schizophrenia spectrum; bipolar disorder; recurrent major depression. Control subjects were randomly selected from the phone book. Patients and controls were carefully matched for age, gender, and educational attainments.

Eligibility criteria for patients were: 18–70 years of age; psychopathological stability at the last clinical evaluation in January−February 2020. Eligible control subjects were 18–70 years of age without psychiatric history. Eligible caregivers were at least 18-year-old first-degree relatives of included patients, spending the majority of the day with the patient. Exclusion criteria were: being diagnosed with COVID-19 or suspected of being infected; being exempt from quarantine for any reason; being hospitalized or having been hospitalized since lockdown imposition; having a severe chronic medical disorder.

All procedures were approved by our Institutional Review Board and in accordance with the Declaration of Helsinki, revised Hong Kong 1989. The study was registered with ClinicalTrials.gov (registration number: NCT04357769) on 22 April 2020. All participants conferred informed consent.

Patients and controls did not differ in age, gender, and educational attainments. Patients with serious mental illness had lower economic status (chi-square: *p* < 0.0001, χ = 46.92) and higher rates of concomitant medical diseases (chi-square: *p* = .001, χ = 11.87). Patients had significantly higher mean scores than non-psychiatric participants on the Perceived Stress Scale (PSS), the Generalized Anxiety Disorder Scale (GAD-7), the Patient Health Questionnaire (PHQ-9), and the Specific Psychotic Experience Questionnaire (SPEQ), Paranoia subscale (Table 1).

**Table 1.** Descriptive statistics and comparisons between groups

	Patients	Controls		Caregivers	
PSS	16.3 ± 8.6	14.1 ± 6.9	*p* = 0.009 *t*_403_ = 2.64 Cohen's *d* = .26	13.6 ± 7.1	n.s.
GAD-7	6.9 ± 5.4	5.5 ± 4.3	*p* = 0.01 *t*_403_ = 2.6 Cohen's *d* = .25	5.3 ± 4.1	n.s.
PHQ-9	9.3 ± 6.2	6.2 ± 4.5	*p* < 0.0001 *t*_403_ = 5.9 Cohen's *d* = .55	4.2 ± 3.2	*p* = 0.003 *t*_250_ = 3.001 Hedges' *g* = .47
SPEQ-Paranoia	10.7 ± 16.3	3.8 ± 7.3	*p* < 0.0001 *t*_403_ = 5.47 Cohen's *d* = .54	2.7 ± 4.8	n.s.
SPEQ-Grandiosity	2.4 ± 3.6	3.2 ±4 .0	*p* = 0.04 *t*_403_ = 2.05 Cohen's *d* = .20	1.5 ± 2.9	*p* = 0.006 *t*_250_ = 2.77 Hedges'*g* = .43
ZBI				20.4 ± 14.4	

n.s., not significant; PSS, Perceived Stress Scale; GAD-7, 7-item Generalized Anxiety Disorder; PHQ-9, 9-item Patient Health Questionnaire; SPEQ, Specific Psychotic Experiences Questionnaire; ZBI, Zarit Burden Interview.

Results were given in mean ± s.d. Independent sample Student's *t* test was used for comparison between patients and controls and between caregivers and controls. The Hedges' *g* test for calculation of effect size was used in the comparison of caregivers and controls due to the disparity in groups' size.

After adjustment, concomitant medical diseases showed an independent effect on PSS, GAD-7, and PHQ-9 score (two-way ANOVA: *p* = .01, *F*_1,384_ = 6.72; *p* = .006, *F*_1,384_ = 7.53; and *p* = .03, *F*_1,384_ = 4.59, respectively). Significant differences in GAD-7 did not survive adjustment for economic status. An effect of economic status was also found on PSS scores in schizophrenia spectrum patients, after subdivision for diagnosis.

Rates of high perceived stress severity (PSS score >26), moderate-severe anxiety (GAD-7 score >10), and severe depressive symptoms (PHQ-9 score >15) were significantly higher in patients v. controls (chi-square: *p* = .001, χ = 13.07; *p* = .013, χ = 8.71; *p* = .001, χ = 13.47, respectively; all survived adjustments). Patients had higher odds of suffering from severe psychopathology compared to controls (logistic regression: *p* = .001, χ = 13.81 OR: 4.23 95% CI 1.8–9.7; *p* = .01, χ = 8.83, OR: 2.14 95% CI 1.2–3.6; and *p* = .001, χ = 13.59 OR 2.5 95% CI 1.5–4.2, respectively). Similar outcomes were also found after subdivision in schizophrenia spectrum and mood disorder patients.

The association between higher risk of moderate−severity anxiety in patients with serious mental illness compared to controls did not survive adjustment for PSS (logistic regression: *p* > 0.05). On the other hand, the association between PSS and patients remained significant even after adjusting for GAD-7 (logistic regression: *p* = 0.04, χ = 6.29).

Surprisingly, caregivers had significantly lower mean PHQ-9 scores and not different PSS or GAD-7 scores compared to controls (Table 1). Mean ZBI score corresponded to mild burden.

These results indicate that patients with serious mental illness had higher levels of COVID-19-related perceived stress, anxiety, and depressive symptoms compared to non-psychiatric participants. Patients were four times more likely to perceive high COVID-19 pandemic-related stress, and had 2–3 times higher risk of severe anxiety and depressive symptoms.

Comparisons with previous reports may suggest that non-psychiatric controls might be experiencing more substantial worsening of psychological distress compared to patients with serious mental illness (Bergomi et al., 2017; Bonfiglio, Renati, Hjemdal, & Friborg, 2016; Gilbody et al., 2019; Lee et al., 2018; Nuyts, Nawrot, Scheers, Nemery, & Casas, 2019; Tso, Grove, & Taylor, 2012).

Nonetheless, it has to be emphasized that the level of distress perceived by patients with serious mental illness due to COVID-19 pandemic and mass quarantine is indubitably higher than that perceived by the general population. Actual perceived stress from COVID-19 outbreak and lockdown restrictions appears a strong predictor and mediator of the heightened risk of suffering from severe anxiety in patients with serious mental illness. They may have higher COVID-19-related perceived stress compared to the general population as a consequence of both their mental illness and higher susceptibility to medical disorders. Uncertain economic status may also play a role.

Despite the global attention focused on the distress in the general population, our findings reinforce the view that the current pandemic might have dramatic consequences for the mental health of patients with serious mental illness.
